# Correction to: 9 papers to add missing data availability statements

**DOI:** 10.1093/oxfimm/iqag005

**Published:** 2026-05-11

This is a correction to the following papers:

https://doi.org/10.1093/oxfimm/iqaa003

https://doi.org/10.1093/oxfimm/iqab012

https://doi.org/10.1093/oxfimm/iqaa006

https://doi.org/10.1093/oxfimm/iqad005

https://doi.org/10.1093/oxfimm/iqad006

https://doi.org/10.1093/oxfimm/iqae010

https://doi.org/10.1093/oxfimm/iqae009

https://doi.org/10.1093/oxfimm/iqaf005

https://doi.org/10.1093/oxfimm/iqag001

In the originally published versions of these papers, the following data availability statement was inadvertently omitted by the authors: “No new data were generated in support of this article.” The statement has been added to the articles DOI iqaf005 and DOI iqag001.

The error is only outlined in this notice for the first seven articles on this list in order to preserve the versions of record.

